# Employing New Hybrid Adaptive Wavelet-Based Transform and Histogram Packing to Improve JP3D Compression of Volumetric Medical Images

**DOI:** 10.3390/e22121385

**Published:** 2020-12-07

**Authors:** Roman Starosolski

**Affiliations:** Department of Algorithmics and Software, Silesian University of Technology, 44-100 Gliwice, Poland; rstarosolski@polsl.pl

**Keywords:** medical imaging, lossless image compression, volumetric medical image compression, hybrid transform, entropy estimation, discrete wavelet transform, reversible denoising and lifting step, histogram packing, JPEG 2000, JP3D

## Abstract

The primary purpose of the reported research was to improve the discrete wavelet transform (DWT)-based JP3D compression of volumetric medical images by applying new methods that were only previously used in the compression of two-dimensional (2D) images. Namely, we applied reversible denoising and lifting steps with step skipping to three-dimensional (3D)-DWT and constructed a hybrid transform that combined 3D-DWT with prediction. We evaluated these methods using a test-set containing images of modalities: Computed Tomography (CT), Magnetic Resonance Imaging (MRI), and Ultrasound (US). They proved effective for 3D data resulting in over two times greater compression ratio improvements than competitive methods. While employing fast entropy estimation of JP3D compression ratio to reduce the cost of image-adaptive parameter selection for the new methods, we found that some MRI images had sparse histograms of intensity levels. We applied the classical histogram packing (HP) and found that, on average, it resulted in greater ratio improvements than the new sophisticated methods and that it could be combined with these new methods to further improve ratios. Finally, we proposed a few practical compression schemes that exploited HP, entropy estimation, and the new methods; on average, they improved the compression ratio by up to about 6.5% at an acceptable cost.

## 1. Introduction

The efficient compression of volumetric medical images is essential for medical picture archiving and communication systems (PACSs), because of the huge amount of such data generated every day in hospitals during routine medical procedures, such as Magnetic Resonance Imaging (MRI) and Computed Tomography (CT) scans. Lossless compression algorithms allow for significantly reducing the size of image files, to the same extent decreasing the demand of PACSs for mass storage capacity and transmission media bandwidth. Although much better compression ratios may be obtained by lossy algorithms, the use of lossy compression for medical images is disputable, guidelines that are issued by various professional bodies recommend different lossy compression ratios, and in some cases lossy compression of such images is forbidden by the law [1,2,3].

This work aimed to improve the lossless compression ratios obtained using the JP3D algorithm for volumetric medical images. JP3D is a part (number 10) of the JPEG 2000 standard designed for compression of three-dimensional (3D) data [4,5,6,7], and, like the entire JPEG 2000, it is based on the discrete wavelet transform (DWT) [8]. The main new contributions of this study consist of an application to 3D image compression of certain new methods that recently were found to be effective for two-dimensional (2D) images. Namely, we applied to 3D-DWT the reversible denoising and lifting steps (RDLS) with step skipping [9] and constructed a hybrid transform that combined 3D-DWT with prediction, like in [10]. We also noticed the significance of histogram packing (HP) [11] in the case of some volumetric images. Finally, we proposed a few low-cost compression schemes, which exploit both the new methods and HP, and that are practical contributions.

The initial phase of the research reported in this paper, which consisted of checking whether improving the DWT-based compression of 3D data was a promising research direction, was presented in a conference report [12]. In that work, we generalized, to the 3D case, two simple fixed 2D-DWT variants that were obtained with the use of step skipping and that were effective for 2D data. Thus, we obtained six fixed 3D-DWT variants and found that, by adaptively selecting among them, the bitrates of medical volumetric images could be improved. In the work reported herein, the 3D-DWT-based transform was constructed for each image individually in a sophisticated way by using the heuristic and entropy estimation. In addition to step skipping, the transform exploited RDLS and hybridization of DWT and prediction (and additionally HP was used).

An evaluation was done using a test-set of medical volumetric images of modalities: CT, MRI, and Ultrasound (US), which has previously been used in [7,12]. The new methods proved effective for 3D data. They resulted in an average compression ratio improvement of up to over 2%, whereas competitive methods that were applied to 3D-DWT (DA-DWT [13,14,15] and JP3D+BP [16]) resulted in an average ratio improvement of less than 1%. Our new methods required an image-adaptive selecting of parameters (like denoising filters for RDLS), which was done by using a heuristic that initially was based on the actual effects of the parameters being selected on the compression ratio. While employing fast entropy estimation of JP3D bitrate in order to reduce the cost of the parameter selection, we found that some MRI images had sparse histograms of intensity levels. Applying HP to these images allowed for effectively using entropy estimation (that had been ineffective without HP) and obtaining significantly better compression ratios. We found that, due to the sparseness of histograms of some images only, the simple and old HP had a greater impact on the average compression ratio of the entire set than the new sophisticated methods (i.e., RDLS with step skipping and the hybrid transform that combines DWT with prediction). Finally, we proposed a few schemes exploiting HP, entropy estimation, and the new sophisticated methods. On average, they improved the compression ratio by up to about 6.5% at a cost acceptable from a practical standpoint.

The remainder of this paper is structured, as follows. In subsections of Section 2, we first describe briefly the 3D-DWT used by lossless JP3D (Section 2.1), and then we present the application of RDLS with step skipping to 3D-DWT (Section 2.2), the hybrid transform combining 3D-DWT with prediction (Section 2.3), and the heuristic for image-adaptive selecting of parameters for the transform exploiting RDLS, step skipping, and prediction (Section 2.4). Next, HP is characterized in Section 2.5, whereas the experimental procedure, test data, and implementations are described in Section 2.6. The experimental results are presented and discussed in Section 3—first, the effects of applying the new methods by using bitrate-based heuristic are investigated (Section 3.1); next, we apply entropy estimation without using HP (Section 3.2) and, in Section 3.3, we combine the new methods, HP, and entropy estimation and analyze their effectiveness with respect to compression ratio and speed, which results in identifying the practical schemes. Section 4 summarizes the findings.

## 2. Materials and Methods

### 2.1. Lifting-Based Discrete Wavelet Transform

DWT is used in image compression algorithms to decompose an image into subbands of different characteristics (subbands represent image details of different orientations and sizes). It is easier to efficiently compress subbands than the original image, because the subbands are less spatially correlated, their entropy is lower, and they have well-defined characteristics that allow for better modeling. There are many additional advantages of decomposition into subbands for lossless and lossy compression, for instance, it allows for various kinds of progressive coding. For brevity, like in the previous works [9,10,12], below we describe only the lifting-based reversible DWT variant with Cohen–Daubechies–Feauveau (5,3) wavelet filter that is exploited in the lossless JP3D, reduced to essentials. For further details and more general characteristics of different variants of DWT, their implementation in JP3D/JPEG 2000 standards, and these standards, the reader is referred to [4,5,6,7,8,17].

The one-dimensional DWT (1D-DWT) transforms a discrete signal $S=s_{0}s_{1}s_{2}\ldots s_{|S|-1}$ of finite length |S| into two subbands:

- a low-pass filtered subband *L* that represents the low-frequency features of *S*; and,
- a high-pass filtered subband *H*, which contains high-frequency signal features that, along with *L*, allow for the perfect reconstruction of *S*.

The transformation of *S* is performed in-place in three below-described steps. First, in the prediction step, the high-pass filtering of odd samples (hereafter, the parity of the sample is determined by its location and not its value) is performed by applying to each odd sample the LS that is presented in Equation (1):

(1) $$s_{x}\leftarrow s_{x}-\left\lfloor \left(s_{x-1}+s_{x+1}\right)/2\right\rfloor .$$

Each LS modifies a single signal sample by adding to it a linear combination of other samples (the sum may be negated). Advantageous properties of a transform that is performed as a sequence of LSs is that it may be computed in-place and it is easily and perfectly invertible. Next, during the update step, another LS (Equation (2)) is applied to each even sample:

(2) $$s_{x}\leftarrow s_{x}+\left\lfloor \left(s_{x-1}+s_{x+1}+2\right)/4\right\rfloor .$$

Finally, in the reordering step, even samples are moved to the lower half of *S*, their ordering is preserved (sample $s_{x}$ is moved to $s_{x/2}$), whereas the odd samples are moved to the upper half; thus, separate subbands *L* and *H*, respectively, are obtained. The reordering step is not an LS.

**Figure 1:**
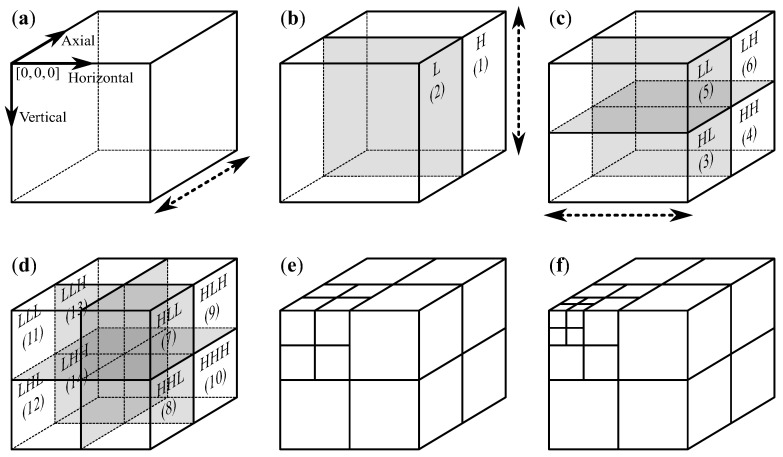
One-level three-dimensional discrete wavelet transform (3D-DWT) (**a**–**d**), two-level 3D-DWT (**e**), and three-level 3D-DWT (**f**); coordinate system presented in panel (**a**), the dotted arrows indicate directions of applying one-dimensional (1D)-DWT, subband ordering numbers (in round brackets) indicate an order of processing subbands by the heuristic from Section 2.4.

In order to obtain the 3D-DWT of a volumetric image, 1D-DWT is first applied in the axial direction, which results in two volumetric subbands *L* and *H* (see Figure 1a–b). Subsequently, 1D-DWT is applied to the volume in the vertical direction (Figure 1c), which produces the LL and HL subbands (obtained from the *L* subband) and LH and HH subbands (obtained from the *H* subband). Finally, 1D-DWT is applied horizontally, which results in the 1-level 3D-DWT of the volume (Figure 1d), which consists of eight subbands: LLL and HLL (obtained from the LL subband), LHL and HHL (from HL), LLH and HLH (from LH), and LHH and HHH (from HH).

The higher-level DWT, which provides multiresolution image representation, is obtained by Mallat decomposition [18]. The l+1-level transform is obtained by applying the one-level transform to the low-pass subband (LLL) of the *l*-level transform (Figure 1e–f).

Not all subbands created while performing the DWT still exist after its completion. Some subbands are further transformed in-place (*L*, *H*, LL, HL, LH, HH, and at all transform levels, except the highest LLL), we will call such subbands the temporary subbands, whereas others will be called the final subbands. The two subbands to which a temporary subband or the original image was transformed by applying a 1D-DWT will be called complementary to each other. For instance, *L* and *H* are complementary, other pairs of complementary subbands are (LL, HL), (LH, HH), (LLL, HLL), (LHL, HHL), (LLH, HLH), and (LHH, HHH).

The task of improving the lossless JP3D bitrates of volumetric medical images is not simple and not many attempts are reported in the literature. Two interesting approaches were evaluated in [7]. In that study, the direction-adaptive DWT (DA-DWT), earlier used for 2D data [13,14,15], was applied to volumetric medical images. Additionally, the other approach, block-based intra-band prediction of DWT transformed subbands (JP3D+BP), was an adaptation of the method that is presented in [16] for 2D images. These approaches improve average bitrates of lossless JP3D for medical volumes by less than 1% at the cost of a high increase in the compression process complexity. We will compare our results with the findings reported in [7] and, for this purpose, we will use the same set of test data.

### 2.2. Reversible Denoising and Lifting Steps and Step Skipping

The sample that is modified by LS (filtered using Equation (1) or Equation (2) in the case of DWT) gets contaminated by noise from the other samples used in this LS, which is an unwanted side effect of LS. In the case of 1D-DWT, noise is propagated to a sample being modified by LS from its two nearest neighbors that are located in the direction in which 1D-DWT is performed; thus, noise gets propagated between subbands. Because, in JP3D, the DWT subbands are encoded independently, noise propagation increases the amount of information that has to be encoded and worsens the bitrates.

RDLS is constructed based on LS by integrating it with denoising filters in order to prevent noise propagation, but preserve the other properties of LS. A surprising property of RDLS is that, despite exploiting the inherently irreversible denoising, it is perfectly reversible. The proertieps of the RDLS approach are more broadly discussed in [9,19]. In RDLS-DWT, the prediction (Equation (1)) and update (Equation (2)) LSs are replaced by RDLSs constructed on their basis, i.e., by:

(3) $$s_{x}\leftarrow s_{x}-\left\lfloor \left(s_{x-1}^{d}+s_{x+1}^{d}\right)/2\right\rfloor \;and$$

(4) $$s_{x}\leftarrow s_{x}+\left\lfloor \left(s_{x-1}^{d}+s_{x+1}^{d}+2\right)/4\right\rfloor ,$$

respectively, where $s_{i}^{d}$ denotes the denoised sample $s_{i}$. Various denosing filters make the RDLS-modified transform more general than the original one. We may turn it into the unmodified transform by using a special denoising filter, denoted None, for which $s_{i}^{d}=s_{i}$ [20]. Another special filter, termed the Null filter, for which $s_{i}^{d}=0$, allows for practically skipping the step [19]. The end of this Section presents other denoising filters used in this research.

In [20], we found that the noise filtering resulted in the best lossless JPEG 2000 bitrate improvements when applied to some RDLS-DWT subbands only, whereas, for some images, the best bitrates were obtained with the entire DWT stage of JPEG 2000 skipped. Thus, we suspected that the optimum might be in-between skipping and applying RDLS-DWT. The prediction and update RDLS-DWT steps may be skipped using the Null filter, which turns them into $s_{x}\;\leftarrow \;s_{x}$. However, the non-lifting reordering step limits the freedom to skip the selected transform parts. In [9], we proposed the SS-DWT and RDLS-SS-DWT that were obtained from DWT and RDLS-DWT, respectively, by allowing skipping any transform computation step, including reordering. The experimental results implied that the reordering step should be skipped if and only if Null was used for both complementary subbands. In comparison to RDLS-DWT, the new transforms resulted in greater bitrate improvements (RDLS-SS-DWT) or similar improvements, but attained at a smaller cost (SS-DWT). Based on analyzing the most frequently skipped steps in SS-DWT, we defined two fixed SS-DWT variants that allowed for a further reduction of the cost of bitrate improvement.

RDLS with step skipping was successfully applied to reversible color space transforms [19,21] (to standard RCT [5], standard YCoCg-R [22], and to two simpler ones [19,23]) and to multiple-level 2D-DWT [9]. It resulted in practically useful improvements of lossless compression ratios for the reversible color space transforms (in the case of standard algorithms JPEG-LS [24,25], JPEG 2000, and JPEG XR [26,27]), and for DWT in the case of JPEG 2000 coding. So far, RDLS and step skipping were only applied to 2D data; in the conference report [12], we obtained preliminary results indicating that these techniques may be useful for volumetric medical images. We generalized the two fixed 2D SS-DWT variants to the 3D case (obtaining six fixed 3D SS-DWT variants) and applied them to medical volumetric images. By adaptively selecting among these variants and the unmodified DWT, we obtained bitrate improvements competitive to the much more complex DA-DWT and JP3D + DWT.

We need 3D denoising filters and a method of their image-adaptive selection to apply RDLS to volumetric data. The latter is described in Section 2.4. The filters that we employ are 3D variants of filters from the earlier research on RDLS. There were four filtering methods used, including three nonlinear filters belonging to a general family of rank-conditioned rank selection filters [28] (see [20] for examples of filtered images):

- Smoothing—a simple low-pass linear averaging filter; the filtered sample is calculated as a weighted arithmetic mean of samples from the window. The weight *w* of the sample in the window center is a parameter of the filter, whereas other samples’ weights are fixed at 1.
- Median—the filtered sample is calculated as a median of samples from the window.
- RCRS-1—this filter replaces a sample with the window median if the sample is greater than or smaller than all other samples in the window.
- RCRS-2—it replaces a sample with the second greatest window sample value if the sample is greater than the median and the greatest; or, if it is smaller than the median and the smallest, it replaces a sample with the second smallest window sample value.

All in all, we use 16 filters: None, Null, five Smoothing filters (one filter with 5×5×5 window and w=1 and four filters with 3×3×3 window and w=1,16,256,and4096), three Median filters (windows 5×5×5, 3×3×3, and 3×3×1), three RCRS-1 filters (window sizes like for Median), and three RCRS-2 filters (window sizes like for Median). The filters were selected after initial checking, which was done by using a greater number of window sizes. Some of the volumes used in this paper have the same resolution in all directions, whereas, for others, a different (lower) resolution is used in the axial direction. For that reason, and because smaller windows mean faster filtering, we tested the window size reduced in the axial direction (3×3×1)—such a window was, in some cases, useful for nonlinear filters, but, for Smoothing filters, the regular hexahedron windows were almost always better.

### 2.3. Hybrid Transform that Combines RDLS-SS-DWT with Prediction

Applying a multidimensional transform to image data is not the only method of making it more compressible. An alternative approach is called the predictive coding. In a predictive algorithm, we use the predictor function in order to guess pixel intensity. The predictor is usually a simple function that uses only a small number of already processed nearest neighbors of the pixel (pixels are visited in a certain order). Next, we calculate the prediction errors (differences between actual and predicted pixels) and encode them instead of encoding pixels. For typical images, the entropy of prediction errors is significantly smaller than the entropy of image pixels. Applying the prediction to the already transformed DWT subbands is not effective (e.g., see [7]). In a typical case, the purpose of prediction (removing spatial correlation, reducing entropy) has already been achieved during the creation of DWT subbands and further significant improvement of the compressibility of such data is not possible. On the other hand, if the Null filter is used in RDLS-SS-DWT, then the resulting subbands may have characteristics that are closer to the untransformed image than to the subbands of the unmodified DWT. Based on the above premise in [10], we proposed a hybrid transform RDLS-SS-DWT+Pred that was obtained by applying prediction to RDLS-SS-DWT final subbands and found that it resulted in a significant improvement of lossless JPEG 2000 compression ratio.

In RDLS-SS-DWT+Pred, to each final subband of RDLS-SS-DWT, a predictor, selected from a set of candidate predictors, is applied that results in the smallest memoryless entropy of prediction errors. One of the candidate predictors (the NOP predictor) predicts that each sample is 0, thus allowing for not using the prediction if the actual predictors increase the subband entropy. The memoryless entropy is computed as:

(5) $$-M\sum_{i=\mathrm{MinPE}}^{\mathrm{MaxPE}}p_{i}\log_{2}p_{i},$$

where *M* denotes the number of samples in the subband, MinPE and MaxPE are the smallest and the greatest prediction errors, respectively, $p_{i}$ is the probability of occurrence of the prediction error *i* in the subband, and we assume that $0\log_{2}0=0$.

In order to apply RDLS-SS-DWT+Pred adaptively to a volumetric image, we need a filter selection heuristic suitable for the three-dimensional transform and for the used entropy estimation-based predictor selection method (see the next section) as well as a set of candidate predictors for volumetric data. Candidate predictors are presented in Table 1, the prediction is performed in a raster scan order (volume slices from front to back, slice rows from top to bottom, pixels in a row from left to right); if a given predictor cannot be computed, a simpler one is used instead (e.g., NOP instead of any other predictor for the front top left pixel).

### 2.4. Heuristic for Adaptive Transform Construction

We construct RDLS-SS-DWT+Pred in an image-adaptive way by selecting an RDLS filter for each subband, including temporary ones. In other words, all RDLSs that were the most recently employed to filter samples of a specific subband use the same filter. Because, even for low transform levels, performing a full search of filters would be too complex (14 filters must be selected for each transform level), we use a greedy heuristic that is based on the NH heuristic that in [10] was effective for 2D images. It consists of the two steps that are presented below (A and B), where step B may be repeated for a given number of iterations:

(A) For each of the denoising filters, check the bitrate that was obtained for an image using this filter for all subbands at all transform levels. Subsequently, for all subbands at all levels, select the filter that results in the best overall bitrate.
(B) For each transform level *a* (starting from level 1) and for each subband *b* (at a specific level analyzed in the order presented in Table 2), try to find a better filter by checking for each filter (except for the one already selected) the bitrate that was obtained using this filter for subband *b* at level *a*, while, for other subbands, the filters selected so far are used; if the Null filter gets selected for a prediction step, then it is also selected for the complementary update step (see Table 2).

Filter selection is based on the filter’s effect on the final bitrate, so it takes into account that, in RDLS-SS-DWT+Pred, the predictor for each final subband is selected by using Equation (5) from a set of candidate predictors and that certain reordering steps are skipped (if for both complementary subbands the Null filter is selected). It should be noted that selecting the Null filter for a prediction step means that it also gets selected for the complementary update step, but the update step’s filter may be later changed again, because a prediction step filter is selected by the heuristic before selecting a filter for its complementary update—see the order of analyzing subbands and their properties in Table 2.

A practical compression method should not be too time-consuming. Employing RDLS-SS-DWT+Pred may slow down the JP3D compressor, because the heuristic selection of denoising filters and predictors requires applying them and testing their effects many times. Testing of each RDLS filter for a given subband requires re-selecting predictors for all the final subbands that are affected by this filter. Additionally, the actual application of the adaptively constructed transform may be more complex than for unmodified DWT. Thus, in this study, we start by assessing the compression ratio improvements attainable by the most complex variants of RDLS-SS-DWT+Pred and then we focus on variants that are useful from a practical standpoint. For the latter, only the Null and None filters are used (i.e., it is the SS-DWT special case of RDLS-SS-DWT); filter selection, instead of being based on the actual JP3D bitrate, employs an estimator of the effects of JP3D entropy coder (as described in Section 2.6) that reuses estimations made while selecting predictors.

For these variants, the computational complexity of the entire compression process involving adaptive transform construction and actual compression consists of: $T_{\mathrm{fs}}$—the cost that is associated with applying prediction and determining the entropy coding effects for the final subbands created during the operation of the heuristic as well as with the final entropy coding of these subbands (Equation (6)), $T_{\mathrm{as}}$—the cost that is associated with applying RDLSs (or just LSs, as we do not use actual denoising filters in SS-DWT) while computing all subbands created by the heuristic, including temporary ones (Equation (7)), and the cost of remaining operations that must be done by the compressor before the transform or after the entropy coding (like image data inputting or the JP3D-compliant bitstream formation and outputting).

(6) $$T_{\mathrm{fs}}=P\left(\left(2+\frac{48}{7}n\left(1-8^{-l}\right)\right)\left(\left(p-1\right)c_{\mathrm{pred}}+pc_{\mathrm{est}}\right)+c_{\mathrm{enc}}\right),$$

where *P* denotes the image size (number of pixels), *n*—the number of iterations of step B of the heuristic, *l*—the transform level, *p*—the number of predictors, $c_{\mathrm{pred}}$—the cost of predicting a sample (average for predictors other than NOP, prediction with NOP is assumed to be costless, NOP is assumed to be in the set of candidate predictors), $c_{\mathrm{est}}$—the cost of estimating the bitrate of a sample, and $c_{\mathrm{enc}}$—the cost of entropy coding of a sample.

(7) $$T_{\mathrm{as}}=P\left(\frac{24}{7}\left(1-8^{-l}\right)+n\left(\frac{1152}{49}-\frac{9}{7}2^{4-3l}l-\frac{9}{49}2^{7-3l}\right)\right)c_{\mathrm{LS}},$$

where $c_{\mathrm{LS}}$ is the cost of a single LS. $T_{\mathrm{as}}$ is an upper complexity bound; this formula assumes that, in step B of the heuristic, the denoising filters that are different than the Null filter are used each time. Note that 3D-DWT is done in $P\frac{24}{7}\left(1-8^{-l}\right)$ LSs.

### 2.5. Histogram Packing

An active intensity level is the intensity level that is actually used by image pixels. In a sparse histogram image, a significant part of the nominally available levels is not active and these levels are located in between the active levels on the image histogram. The histogram level utilization *U* is a simple measure of histogram sparseness:

(8) $$U=\frac{L}{1+\mathrm{MaxL}-\mathrm{MinL}},$$

where MaxL and MinL are the highest and the lowest active levels, respectively, and *L* is the number of active levels. 0<U≤1, the smaller the *U*, the more sparse the histogram is.

The adverse effect of histogram sparseness on lossless compression ratios of natural and medical 2D images has been well known for over a decade [11,29]. Applying HP to sparse histogram images before regular compression leads to significant ratio improvements. HP simply maps all of the active intensity levels to the lowest part of the nominal intensity range by using an order-preserving one-to-one mapping. Although, recently, there have been a few publications describing the effective use of HP in lossless image compression (e.g., see [30,31,32]), it seems almost forgotten now. Interestingly, HP has also been used in lossy image compression [33,34].

HP requires the information permitting restoration of the original histogram to be stored with the compressed image. Many methods of encoding this information are available [29]; in this research we will use the Bit-Array that, for each nominally available level, encodes on a single bit whether the level is active. The overhead due to Bit-Array is negligible (32 bytes for 256 nominal levels of an 8-bit image and 512 bytes in the case of an image of 12-bit depth).

### 2.6. Procedure

We used the IRIS-JP3D version 1.1.1 reference implementation of the JP3D standard, developed by Tim Bruylants from Vrije Universiteit Brussel (VUB) and Interdisciplinary Institute for BroadBand Technology (IBBT) [7] in which we modified the DWT stage; our implementation of RDLS-SS-DWT+Pred is available [35] as a patch to IRIS-JP3D. Except for setting the transform variant and the decomposition level (the three-level decomposition was used in all experiments), the default compressor parameters were used. When measuring the compression time, we additionally used a larger than default packet size ($2^{6}\times 2^{6}\times 2^{4}$ instead of $2^{4}\times 2^{4}\times 2^{4}$), because, in IRIS-JP3D, the creation of standard size packets was excessively slow for volumetric images. It is noteworthy that the packets to be placed in the JP3D codestream are created from the already entropy encoded subbands (this JP3D compression stage follows the DWT transform and entropy coding stages). The compression time was measured on a computer that was equipped with an Intel i7-8550U CPU (clock speed 3.96 GHz) and 16 GB RAM, the compressor executable was compiled with Visual Studio Enterprise, version 16.4.3, as a single-threaded application for the x64 target platform.

The compression ratio or bitrate *r*, expressed in bits per pixel (bpp), is calculated using the total size in bytes of the compressed image, including the compressed file format header, the RDLS-SS-DWT+Pred parameters (RDLS filters, predictors), and the original histogram (encoded as BitArray). The bitrate is directly proportional to the compressed file size; hence, a smaller bitrate means a better compression result. The effects of the RDLS, step skipping, prediction, and HP on the JP3D bitrate were analyzed based on bitrate changes with respect to the reference bitrate obtained with unmodified IRIS-JP3D. The bitrate change Δr was expressed in percentage of the reference bitrate.

The heuristic may select RDLS filters by testing their effect on the JP3D bitrate either using an actual entropy coder or a much faster estimation of the coding effects. We use both approaches; as the estimator, we employ the memoryless entropy H0 of the image transformed using *l*-level RDLS-SS-DWT+Pred. H0 is calculated as a sum of memoryless entropies (Equation (5)) of all subbands that would be independently encoded by JP3D with an unmodified *l*-level DWT, regardless of the skipped reordering steps (for instance, 22 subbands for three-level transform). H0 imitates the behavior of the entropy coder of the actual implementation we used. We modified only the DWT stage of IRIS-JP3D; if some reordering steps are skipped, then the entropy coding stage, which is unaware of the skipped reordering steps, may encode a single RDLS-SS-DWT+Pred subband as 2 or more separate subbands that would be created if the reordering steps were not skipped. An important advantage of this estimator is that it may be calculated using the entropies of the predicted final subbands that have already been computed by the heuristic. H0 is only employed for the image-adaptive construction of RDLS-SS-DWT+Pred; the bitrates and bitrate changes in each case are calculated based on the actual compressed image file sizes.

We used the test-set of medical volumetric images that was earlier used in [7,12] for the evaluation of other methods of improving JP3D; it was made available to us thanks to the courtesy of Tim Bruylants. The set is described in detail in [7], it contains 11 images of the following modalities:

- Computed Tomography (CT)—six scans (CT1...CT6) of 12-bit depth and sizes (width × height × depth, in pixels) from 512 × 512 × 44 to 512 × 512 × 672,
- Magnetic Resonance Imaging (MRI)—three scans (MRI1...MRI3) of 12-bit depth and sizes from 256 × 256 × 100 to 432 × 432 × 250, and
- Ultrasound—two volumes (US1 and US2) of 8-bit depth and sizes 500 × 244 × 201 and 352 × 242 × 136, respectively.

## 3. Results and Discussion

### 3.1. Application of RDLS with Step Skipping and Prediction to Volumetric Data

In Table 3, we present the bitrates that were obtained for volumetric images from our test-set by using JP3D with unmodified DWT (DWT bitrate) and the bitrate changes obtained by modifying the DWT stage of JP3D. The following modifications were investigated: RDLS-SS-DWT, SS-DWT, as well as the hybrid transforms that were obtained by applying prediction to subbands produced by RDLS-SS-DWT, SS-DWT, and to the otherwise unmodified DWT (denoted RDLS-SS-DWT+Pred, SS-DWT+Pred, and DWT+Pred, respectively). Denoising filters for RDLS and DWT steps to be skipped were selected by the heuristic based on actual JP3D bitrates.

RDLS with step skipping improved the bitrates of all volumes in the test-set. The average improvement due to RDLS-SS-DWT of approximately 1.39% is not great from a practical standpoint. However, this result is quite good when compared to a couple of other methods of improving JP3D bitrate. During the initial research phase [12] by adaptively selecting fixed SS-DWT variants for these images, the bitrate was improved by 0.59% on average. In [7], for the same set of volumetric medical images that are used in this paper, two more complex modifications of 3D variants of DWT and JP3D were investigated: the DA-DWT and JP3D+BP mentioned in Section 1. The latter one was more effective and it resulted in an improvement of the average bitrate by 0.82%. The majority of the bitrate improvement of RDLS-SS-DWT is obtained with the much simpler SS-DWT, which is worse by 0.12 percentage points only. In the next Section, we use this observation to find a low-complexity variant of our method.

Looking at the results of hybrid schemes that combine prediction with DWT, we see that the prediction is not effective when applied to DWT without RDLS and step skipping, but, when combined with RDLS-SS-DWT or SS-DWT, it allows for greater bitrate improvements of about 2% on average. The improvement is mainly caused by much better bitrates of hybrid schemes for the MRI1 image. For other images, the bitrate improvement is also better on average, but not for every image, which may be attributed to the heuristic method of transform construction. The difference between bitrate improvements of RDLS-SS-DWT+Pred and SS-DWT+Pred is very small, which indicates that using actual denoising filters in RDLS (filters different than Null and None, which are time-consuming to apply and to select by the heuristic) may be avoided without sacrificing bitrate improvements.

### 3.2. Employing Entropy Estimation for Selection of Skipped Steps

Entropy coding is the slowest part of JP3D. Instead of using the actual JP3D bitrate, an estimated bitrate may be used, in order to reduce the cost of the heuristic construction of SS-DWT. Such an approach proved to be effective for 2D images in the cases of SS-DWT, RDLS-SS-DWT, and their hybrid variants exploiting prediction [9,10]. In Table 4, we report the JP3D bitrate changes that were obtained when for selection of SS-DWT and SS-DWT+Pred steps to be skipped, H0 was used instead of the actual bitrate; these variants are denoted SS-DWT(H0) and SS-DWT(H0)+Pred, respectively.

The use of entropy estimation in the heuristic deteriorates the results on average. The bitrate of non-hybrid transform SS-DWT(H0) is even worse than in the case of the unmodified DWT. However, except for the MRI1 (only in the case of SS-DWT(H0)) and MRI3 images, entropy estimation only slightly decreases the improvement in the compression ratio (by 0.17 percentage points on average). A closer look at the characteristics of the images has identified the cause of worse estimation results. The MRI1 and MRI3 images have sparse histograms, their histogram level utilization *U* is 19% and 35%, respectively, whereas, for other images, *U* is from 65% to 100%. Our simple memoryless entropy-based compression effect estimation becomes imprecise on such data. Transforms, such as DWT or prediction, increase the entropy of sparse histogram data. Thus, by applying histogram packing, not only the estimation quality, but also the attainable compression ratios may be significantly improved, which is investigated in the next section.

### 3.3. Practical Schemes Exploiting Histogram Packing

Because HP may have a significant impact on the compression results, first in Table 5 we report the compression ratio improvements attainable by using HP with unmodified DWT (HP+DWT) and with some of the earlier investigated variants of DWT that were constructed by a heuristic exploiting actual JP3D bitrates. Employing HP is denoted by adding the prefix “HP+” to the variant name. Next, in Table 6, we check whether entropy estimation may be used by the heuristic for histogram-packed volumetric images without causing the bitrate deterioration that was observed for sparse-histogram images.

The use of HP in conjunction with all other DWT improvement methods studied earlier in this article (HP+RDLS-SS-DWT+Pred) resulted in an average compression ratio improvement of 6.66%. This is a very good result when compared to competitive methods and it is a significant improvement of the bitrate of a lossless algorithm. Although the original aim of the research was to exploit the new sophisticated methods in JP3D, the simplest HP that is known for a long time proved to be the most effective. Combining HP with DWT (HP+DWT), we obtain an average improvement of 5.34%. This way, for sparse histogram images MRI1 and MRI3, the JP3D compression ratio gets improved by over 20%, but the bitrates of other images are not significantly affected. For improving the bitrates of the latter images, we also have to apply at least some of the new methods.

It is worth noting that part 2 of the JPEG 2000 standard [17], among other extensions of the baseline JPEG 2000, defines in its Annex K the non-linear transformation (NLT). NLT may be used to restore, within the standard JPEG 2000 pipeline, the original histogram of the image data that was subjected to HP. Part 10 of the standard, which defines JP3D, explicitly allows for the use of NLT. Thus, the use of HP is compliant with JP3D, i.e., it does not require modifying the part 10-compliant decoder, provided that it supports this extension. Specific JPEG 2000 implementations support the baseline standard (part 1) and they may support selected further parts and extensions of the standard; the IRIS-JP3D implementation that we used did not support NLT.

The difference between the effects of HP+RDLS-SS-DWT+Pred and HP+SS-DWT+Pred is similarly small as without HP. From a practical point of view, the additional cost of using actual denoising filters is not justified. These filters do not significantly improve the bitrate, although they are used by HP+RDLS-SS-DWT+Pred (by this transform, filters that were different than Null and None were used for about 25% of the subbands). Prediction has a slightly larger impact; its use improves the HP+SS-DWT bitrate by almost 0.1 percentage points. The effect of using prediction is not big, so, in order to check whether this transform indeed is a hybrid combination of DWT with prediction, we tested how often the actual prediction was applied to the final subbands of HP+SS-DWT+Pred. Indeed, it is a hybrid transform; predictors other than NOP are used for about one-third of the final subbands (the most often used is the MED_XY predictor, followed by AVG3D and P_X). To some extent, the use of prediction and RDLS with step skipping allowed for adapting the DWT transform to the inadequate data characteristic, but much better results were obtained by improving the characteristic of the data by using HP.

**Figure 3:**
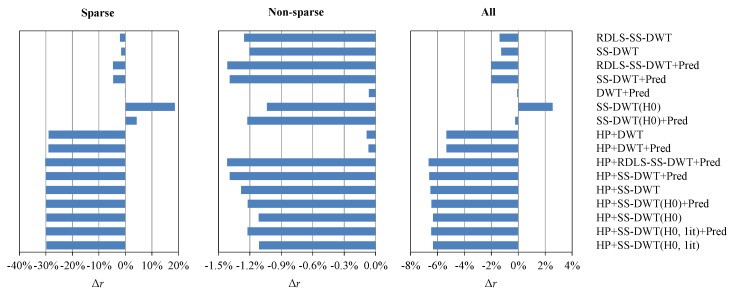
Average bitrate changes due to applying transform variants from Table 3, Table 4, Table 5 and Table 6 to sparse- histogram images, non-sparse histogram images, and for all images from the set.

In Table 6, we report the JP3D bitrate changes due to combining HP with entropy estimation-based SS-DWT variants from Table 4. Additionally included are variants that were obtained by reducing the number of iterations of step B of the heuristic from the default two to one iteration; these variants are denoted by “(H0, 1it)”. They were included, because, in the case of 2D photographic images, using one iteration of a similar heuristic was sufficient [10]. Figure 3 presents the average JP3D bitrate improvements that were obtained for the sparse histogram images (i.e., MRI1 and MRI3), average improvements for other images, and average improvements for the entire test-set; reported are the improvements that were obtained by all of the transform variants from Table 3, Table 4, Table 5 and Table 6 (i.e., all variants that use or not use HP and use or not use the entropy estimation).

We can see that the use of HP has a very positive impact on the effects of entropy estimation of JP3D bitrate used by the heuristic. Bitrate improvements are close to those that were obtained when the actual JP3D bitrate was used for the selection of HP+SS-DWT and HP+SS-DWT+Pred steps to be skipped. Exploiting only one iteration of step B of the heuristic is sufficient, when compared to using two iterations; it does not significantly affect the bitrate improvement (it may even lead to a better average bitrate). The difference between the results that were obtained by using only HP and SS-DWT, and the results that were obtained by additionally using the remaining investigated methods (prediction, RDLS with actual denoising filters, and using the estimated bitrate in heuristic instead of an actual one) is negligible for sparse histogram images but noticeable for images having non-sparse histograms. Thus, especially in the case of the latter, the cost of applying the variant may decide, in practice, which variant we should use.

In Table 7, we report, for the transform variants from Table 5 and Table 6, the total compression time (including the histogram packing, heuristic, entropy coding, etc.) relative to the unmodified JP3D. E.g., the relative time equal to 1.47 means that the compression takes 47% more time than in the case of the unmodified JP3D. This time was estimated by using the actual execution times of the elements of JP3D and the proposed modifications, averaged for all images in the test-set (Table 8) and Equations (6) and (7) (or similar formulas for variants from Table 5 that do not use entropy estimation for selecting of steps to be skipped). For time estimation, we conservatively assumed that predictors other than NOP were equally as complex as MED_XY, which is actually one of the most complex predictors used.

The HP+SS-DWT(H0, 1it) variant is the best practical trade-off in most cases. At the cost of increasing the compression time by less than half, it obtains an average bitrate improvement of approximately 6.33%, which is quite close to the maximum improvement that was obtained in this study (6.66% by using HP+RDLS-SS-DWT+Pred). This variant is based on entropy estimation and it only uses the two simplest modifications that we tested, i.e., HP and step skipping. The cost of extending this variant with the prediction (HP+SS-DWT(H0, 1it)+Pred) may be acceptable in practice. However, only in the case of non-sparse images (see Figure 3), the further bitrate improvement obtained with HP+SS-DWT(H0, 1it)+Pred is a noticeable part of the improvement of the simpler HP+SS-DWT(H0, 1it). The HP+SS-DWT and HP+SS-DWT+Pred transform variants constructed by the heuristic using bitrate to select the steps to be skipped (including variants exploiting only one iteration of step B of the heuristic that are not presented in Table 7) result in compression speeds that are roughly three to six times slower than in the case of their entropy estimation-based counterparts. Even more expensive is the use of actual denoising filters in the HP+RDLS-SS-DWT+Pred transform, which further reduces the compression speed by an order of magnitude. Much more time is required for HP+RDLS-SS-DWT+Pred, because of the large number of actual denoising filters used in RDLSs (14) and the fact that the cost of the more complex ones, such as the median filter with a large window (5 × 5 × 5 pixels), can be greater than the cost of entropy coding.

We also note that significantly better compression ratios of volumetric medical images may be obtained using compression algorithms that employ different methods than DWT. Probably, the best bitrates are obtained by the 3D-MRP algorithm [36], which is based on the Multiple Rate Predictors (MRP) algorithm that was proposed for 2D images [37,38]. As reported in [36], for a set of 8-bit CT and MRI volumetric images and a set of 16-bit CT and MRI volumetric images, 3D-MRP allowed for obtaining the average bitrate smaller than JP3D by as much as 50.4% and 14.7%, respectively. However, the compression time was roughly 6000 times longer than in the case of JP3D. A faster variant of 3D-MRP obtained bitrates that were smaller than JP3D by 47.3% and 14.2%, respectively, while being over 800 times slower. As found in [30] for 12- and 16-bit sparse histogram volumetric images, HP also improves the compression ratios of MRP-based algorithms.

## 4. Conclusions

The primary purpose of the reported research was to improve the DWT-based JP3D compression of volumetric medical images by applying new methods that were previously only used in the compression of 2D images. Namely, we applied RDLS with step skipping to 3D-DWT and constructed a hybrid transform that combined 3D-DWT with prediction. The performance was evaluated using a test-set of medical volumetric images of modalities: CT, MRI, and US. The hybrid transform proved effective thanks to RDLS with step skipping. The greatest ratio improvement (1.3% on average) was due to the use of step skipping, the hybrid combining of prediction with SS-DWT increased the improvement to almost 2%, whereas the use of RDLS with actual denoising filters had a much lower impact (increased the improvement to slightly more than 2%). Furthermore, the new methods resulted in improving the compression ratios to a greater extent than the competitive methods DA-DWT and JP3D+BP.

The heuristic image-adaptive selection of transform parameters (denoising filers, steps to be skipped, and predictors) based on the actual bitrate of the compression algorithm is expensive. In order to obtain a practical compression scheme, we employed a fast entropy estimation using *H*0 to select the parameters, but the initial estimation results were unsatisfactory. Analyzing the cause, we found that some MRI images had sparse histograms and, although only in their case, the estimation led to a compression ratio deterioration, the deterioration was so significant that it thwarted the average improvement for the entire set. Therefore, we applied HP, which significantly improved the compression ratios of sparse histogram images. The average ratio improvement for these images only due to HP (without using RDLS, step skipping, or prediction) was almost 29%; consequently, the improvement for the entire set only due to HP was about 5.3%. This is an important result—the significance of histogram sparseness seems almost forgotten nowadays, while some volumetric images are sparse and exploiting HP can have a much greater impact on compression effects than using sophisticated new methods. Furthermore, the cost of applying HP is negligible and the use of HP is compliant with JP3D. Our new methods combined with HP allow further improvements for sparse histogram images; using them all (HP+RDLS-SS-DWT+Pred), an average improvement of over 30% for sparse histogram images and approximately 6.6% for the entire set was obtained. HP made entropy estimation effective for all images and allowed for us to propose practical variants with a reduced cost.

All in all, among the investigated variants, the most useful practical tradeoffs appear to be HP+DWT, HP+SS-DWT(H0, 1it)+Pred, and HP+SS-DWT(H0, 1it). The simplest HP+DWT obtains, in the average case, the majority of the improvement possible with the most complex variants at the cost of reducing the compression speed by a quarter percent, but it is the result of only improving the ratios of sparse histogram images. The use of HP+SS-DWT(H0, 1it)+Pred or the simpler HP+SS-DWT(H0, 1it) gives good results for all images at an acceptable cost of increasing the compression time by approximately 160% or less than 50%, respectively. The ratio improvement obtained in this way is large for sparse histogram images (about 30%); for others, it exceeds 1.2% and 1.1%, respectively; the compression ratio improvement for the entire set is about 6.5% and 6.3%, respectively.

A promising direction of further research is the use of the detector precision characteristic (DPC) method [39], which allows for a virtually costless adaptive construction of the transform based on a model that is driven by image acquisition parameters, which are normally stored along with medical volumes. We have already obtained positive results by employing DPC to adaptively select denoising filters for RDLS-modified color space transforms [21]. Furthermore, we suspect that RDLS effects could be improved by using sophisticated denoising filters, which, in conjunction with the adaptive DPC-based method of their selection, may allow the most sophisticated hybrid transform HP+RDLS-SS-DWT+Pred to obtain greater compression ratio improvements at an acceptably low cost.

## Figures and Tables

**Figure 2:**
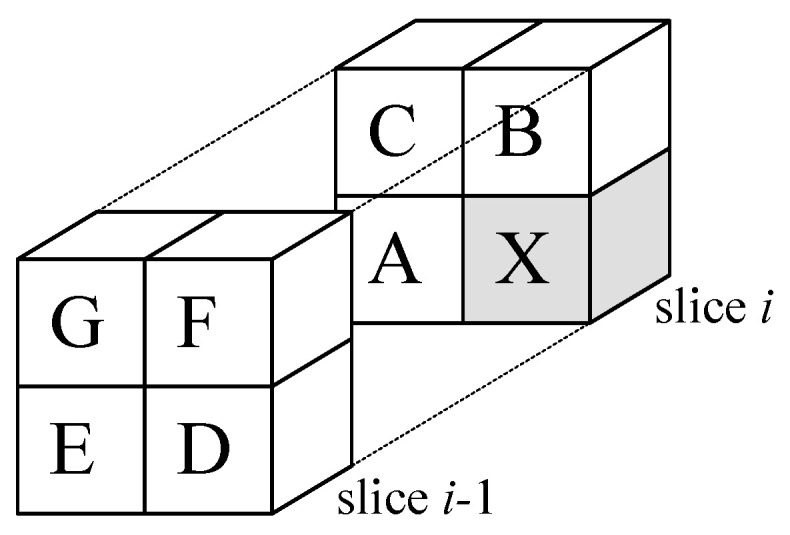
Locations of neighboring pixels used by predictors from Table 1; X—the pixel being predicted.

**Table 1:** Candidate predictors.

Predictor	Prediction
NOP	0
P_X	A
P_Y	B
P_Z	D
AVG_XY	(A+B)/2
AVG_XZ	(A+D)/2
AVG_YZ	(B+D)/2
MED_XY	median(A,B,A+B−C)
MED_XZ	median(A,D,A+D−E)
MED_YZ	median(B,D,B+D−F)
AVG3D	(A+B+D)/3

Note: neighbors’ locations presented in Figure 2.

**Table 2:** Order of analyzing subbands by the heuristic and properties of subbands.

Ordering Number	Subband	Step Type	Final or Temporary	Complementary to Subband
1	*H*	prediction	temporary	*L*
2	*L*	update	temporary	*H*
3	HL	prediction	temporary	LL
4	HH	prediction	temporary	LH
5	LL	update	temporary	HL
6	LH	update	temporary	HH
7	HLL	prediction	final	LLL
8	HHL	prediction	final	LHL
9	HLH	prediction	final	LLH
10	HHH	prediction	final	LHH
11	LLL	update	varies ${}^{*}$	HLL
12	LHL	update	final	HHL
13	LLH	update	final	HLH
14	LHH	update	final	HHH

*—final at the highest transfom level, temporary at other levels.

**Table 3:** JP3D bitrate changes Δr due to reversible denoising and lifting steps (RDLS), step skipping, and prediction.

Image	DWT Bitrate *r* (bpp)	RDLS-SS-DWT	SS-DWT	RDLS-SS-DWT +Pred	SS-DWT +Pred	DWT +Pred
CT1	4.911	−0.684%	−0.618%	−0.573%	−0.528%	0.214%
CT2	7.632	−0.105%	−0.063%	−0.175%	−0.148%	−0.063%
CT3	5.437	−0.558%	−0.465%	−0.497%	−0.397%	0.003%
CT4	3.844	−1.995%	−1.943%	−2.234%	−2.218%	−0.222%
CT5	2.822	−3.083%	−3.006%	−3.913%	−3.898%	−0.058%
CT6	5.029	−0.791%	−0.765%	−1.252%	−1.246%	−0.122%
MRI1	3.503	−3.712%	−2.983%	−8.973%	−8.973%	−0.271%
MRI2	4.091	−1.790%	−1.727%	−1.777%	−1.796%	−0.301%
MRI3	6.588	−0.304%	−0.194%	−0.384%	−0.324%	−0.091%
US1	4.840	−1.279%	−1.265%	−1.300%	−1.288%	−0.021%
US2	5.233	−1.002%	−0.992%	−1.014%	−1.004%	−0.012%
Average	4.903	−1.391%	−1.275%	−2.008%	−1.984%	−0.086%

Note: The reference bitrate of JP3D with unmodified DWT is expressed in bpp, whereas the bitrate changes due to introducing transform variants are expressed in percentages of the reference bitrate.

**Table 4:** JP3D bitrate changes Δr obtained with the use of entropy estimation-based heuristic.

Image	SS-DWT(H0)	SS-DWT(H0) +Pred
CT1	−0.426%	−0.119%
CT2	0.021%	−0.056%
CT3	−0.415%	0.289%
CT4	−1.704%	−1.978%
CT5	−2.726%	−4.232%
CT6	−0.707%	−1.187%
MRI1	−0.815%	−8.973%
MRI2	−1.321%	−1.634%
MRI3	38.095%	17.510%
US1	−1.072%	−1.095%
US2	−0.987%	−1.000%
Average	2.540%	−0.225%

**Table 5:** JP3D bitrate changes Δr obtained with the use of histogram packing (HP) and the new methods.

Image	HP+ DWT	HP+ DWT +Pred	HP+ RDLS-SS-DWT +Pred	HP+ SS-DWT +Pred	HP+ SS-DWT
CT1	−0.072%	0.214%	−0.573%	−0.528%	−0.689%
CT2	−0.020%	−0.064%	−0.176%	−0.148%	−0.082%
CT3	−0.024%	0.001%	−0.498%	−0.399%	−0.479%
CT4	−0.140%	−0.249%	−2.241%	−2.226%	−2.060%
CT5	−0.187%	−0.058%	−3.914%	−3.898%	−3.178%
CT6	−0.107%	−0.122%	−1.253%	−1.247%	−0.863%
MRI1	−37.139%	−37.266%	−39.270%	−39.143%	−39.232%
MRI2	−0.220%	−0.301%	−1.778%	−1.796%	−1.953%
MRI3	−20.804%	−20.865%	−21.202%	−21.127%	−21.024%
US1	0.000%	−0.021%	−1.300%	−1.288%	−1.264%
US2	0.000%	−0.012%	−1.014%	−1.004%	−0.992%
Average	−5.337%	−5.340%	−6.656%	−6.619%	−6.529%

**Table 6:** JP3D bitrate changes Δr due to HP and new methods with entropy estimation-based heuristic.

Image	HP+ SS-DWT(H0) +Pred	HP+ SS-DWT(H0)	HP+ SS-DWT(H0, 1it) +Pred	HP+ SS-DWT(H0, 1it)
CT1	−0.119%	−0.487%	−0.119%	−0.487%
CT2	−0.027%	−0.003%	−0.027%	−0.003%
CT3	0.288%	−0.426%	0.288%	−0.426%
CT4	−1.987%	−1.823%	−1.943%	−1.823%
CT5	−4.224%	−2.894%	−4.224%	−2.842%
CT6	−1.188%	−0.802%	−1.247%	−0.817%
MRI1	−38.806%	−39.080%	−39.101%	−39.081%
MRI2	−1.635%	−1.548%	−1.625%	−1.548%
MRI3	−21.114%	−20.545%	−21.112%	−20.545%
US1	−1.095%	−1.072%	−1.095%	−1.072%
US2	−1.000%	−0.987%	−1.000%	−0.987%
Average	−6.446%	−6.333%	−6.473%	−6.330%

**Table 7:** Compression time relative to unmodified JP3D.

Transform Variant	Relative Time
HP+DWT	1.00
HP+DWT+Pred	1.14
HP+RDLS-SS-DWT+Pred	>100.00
HP+SS-DWT+Pred	14.02
HP+SS-DWT	11.95
HP+SS-DWT(H0)+Pred	4.00
HP+SS-DWT(H0)	1.94
HP+SS-DWT(H0, 1it)+Pred	2.64
HP+SS-DWT(H0, 1it)	1.47

**Table 8:** Average execution times of the elements of JP3D and proposed modifications.

Element of the Compression Process	Time (ms per 10^6^ pixels)	Percentage of Unmodified JP3D
Unmodified JP3D	307.35	100.00%
3-level 3D-DWT	20.30	6.60%
Entropy coding	209.22	68.07%
Remaining JP3D operations	77.84	25.33%
Entropy estimation	0.57	0.19%
Perdiction (MED_XY)	3.47	1.13%
HP	0.77	0.25%
